# Adaptive Digital Marketing: A Systematic Review of Bio-Inspired Reinforcement Learning, Multi-Agent Systems, and Agentic AI for Intelligent Optimisation

**DOI:** 10.3390/biomimetics11070476

**Published:** 2026-07-08

**Authors:** Tek Narayan Adhikari, William Sayers, Shujun Zhang

**Affiliations:** School of Business, Computing and Social Sciences, University of Gloucestershire, The Park, Cheltenham GL50 2RH, UK; tekadhikari@connect.glos.ac.uk (T.N.A.); szhang@glos.ac.uk (S.Z.)

**Keywords:** bio-inspired computation, reinforcement learning, multi-agent reinforcement learning, agentic artificial intelligence, digital marketing optimisation, swarm intelligence, foraging theory, ant colony optimisation, systematic review, real-time bidding

## Abstract

Background: Digital marketing increasingly functions as a complex adaptive system characterised by non-stationary environments, strategic interaction, and multi-agent competition. Programmatic advertising exemplifies this complexity, where decisions must be made in real time under uncertainty. Under such conditions, traditional static optimisation methods often fail to deliver robust performance. This review synthesises bio-inspired computational approaches, reinforcement learning (RL), multi-agent reinforcement learning (MARL), and agentic artificial intelligence (AI) to develop an integrated theoretical perspective on adaptive optimisation in digital marketing. Methods: Following PRISMA 2020 guidelines, we conducted a systematic search of peer-reviewed research across six databases: Scopus, IEEE Xplore, ACM Digital Library, SpringerLink, ScienceDirect, and arXiv, supplemented by manual reference checking. Each computational paradigm is explicitly grounded in foundational biological literature, including work on evolution, foraging, swarm intelligence, and immune cognition. Reinforcement learning supports adaptive decision-making through mechanisms closely aligned with operant conditioning and foraging behaviour. Multi-agent reinforcement learning extends these principles to interactive marketing ecosystems via decentralised coordination and swarm-based learning. Agentic AI further advances adaptive capability by introducing goal-directed reasoning, memory, and higher-level decision orchestration. Contributions: The review identifies persistent fragmentation across marketing sub-domains and a lack of formal mathematical grounding for widely used bio-inspired analogies. To address these gaps, the study proposes a multi-layer bio-inspired framework and outlines a structured research agenda to guide the development of autonomous digital marketing systems.

## 1. Introduction

### 1.1. Digital Marketing as a Complex Adaptive System

Digital marketing today operates as a complex adaptive system (CAS), shaped by distributed decision-making, non-linear dynamics, continuous feedback loops, and emergent behaviour patterns [1,2]. Real-time bidding (RTB) markets operate at extremely high speeds, often requiring advertisers to make bidding decisions within milliseconds [3,4,5]. Alongside these dynamics, frequent updates to platform algorithms, shifts in consumer behaviour, and the continuous adaptation of competitor strategies produce an environment that remains non-stationary over time, thereby undermining the foundational assumptions of static optimisation techniques [6,7,8]. Large-scale personalisation requires the simultaneous optimisation of decisions across millions of heterogeneous users [5,9,10]. Taken together, these requirements give rise to a dynamic optimisation landscape closely analogous to a biological ecosystem in which multiple agents compete for limited resources under conditions of uncertainty [1,11,12,13].

### 1.2. Limitations of Classical Optimisation

Traditional marketing optimisation approaches are grounded in simplifying assumptions, most notably stationarity, independence among decision-making agents, and stable reward structures [5,8,14]. Formally, classical reinforcement learning and optimisation frameworks assume stationary reward distributions, independent decision-makers, and time-separable objectives [5]. However, real-world digital marketing environments systematically violate these assumptions through endogenous reward interdependencies, policy-dependent state transitions, and delayed or ambiguous credit assignment [6,7,15,16]. As a result, static optimisation methods become provably suboptimal under conditions of non-stationarity and strategic interaction [5,7,15,17]. These limitations motivate the adoption of bio-inspired adaptive paradigms, including reinforcement learning, multi-agent reinforcement learning, and agentic AI frameworks grounded in biological evolutionary, swarm, and cognitive principles [1,2,18,19,20,21].

### 1.3. Bio-Inspired Computation as a Theoretical Lens

This section establishes the conceptual foundation of the review by mapping established biological principles onto the three computational paradigms examined in this study. The purpose is to justify the use of bio-inspired analogies as interpretive lenses and to situate the review within a coherent theoretical framework. Biological systems provide well-established models of how distributed optimisation can be achieved under dynamic and uncertain conditions. Operant conditioning corresponds with reinforcement learning, where behaviour is shaped through reward-driven feedback and iterative updating [5,22,23]. Foraging theory, formalised by Stephens and Krebs [11], offers a principled account of how organisms balance exploration and exploitation—a logic that underlies a range of modern sequential decision-making strategies [24,25,26,27,28]. At a collective level, swarm intelligence demonstrates how coordination and adaptation can emerge from local interactions without central control, providing conceptual grounding for MARL architectures [2,12,19]. Co-evolutionary processes studied in evolutionary computation capture how competing agents adapt in response to one another [1,18]. Finally, immune-inspired cognitive models offer mechanisms for memory, planning, and anomaly detection, informing the design of agentic AI systems [13]. These biologically grounded principles represent functional correspondences that can inform computational models for adaptive marketing systems.

### 1.4. Research Gap and Contribution

Although RL, MARL, and agentic AI have been studied independently, no unified systematic synthesis explicitly links these paradigms within a bio-inspired framework for digital marketing. This paper contributes (1) a multi-layer bio-inspired framework linking biological principles to computational paradigms and marketing capabilities; (2) a systematic synthesis of relevant studies; (3) the identification of two critical research gaps based on the reviewed literature; and (4) a structured research agenda for autonomous marketing systems.

### 1.5. Research Questions

- RQ1: How are bio-inspired RL methods designed and applied for adaptive optimisation in digital marketing?
- RQ2: How does MARL model coordinate and co-adapt in marketing ecosystems?
- RQ3: How does agentic AI enable strategic autonomy and orchestration in adaptive marketing systems?

## 2. Theoretical Background and Bio-Inspired Foundations

### 2.1. Core Biological Principles

Biological systems demonstrate adaptive intelligence through five interrelated principles that underpin this review, each defined here with a biological example and an explicit computational counterpart. Self-organisation enables decentralised entities to coordinate locally without central control [19], as observed in ant colonies constructing complex structures through purely local interactions. Emergence captures how simple local interactions yield complex system-level outcomes, such as the coherent movement of bird flocks. Co-evolution [18] parallels competing advertisers adjusting strategies in response to rivals, where the fitness landscape of each agent is partially defined by the strategies of others. Resilience [13] reflects the ability of systems to withstand shocks—directly applicable to marketing systems facing platform updates and market disruptions. Finally, foraging optimality [11] formalises how organisms maximise energy intake under uncertainty, providing the foundational biological basis for exploration–exploitation trade-offs in RL. These five principles are not merely metaphorical; they each have formal computational counterparts examined in Section 4, Section 5 and Section 6.

### 2.2. Reinforcement Learning as Operant Conditioning

Reinforcement learning (RL) formalises trial-and-error learning to maximise cumulative reward [5]. The process is conceptually aligned with operant conditioning, where behaviour is shaped through reward-driven feedback. Theoretical foundations trace to Q-learning [14], policy gradients [23,29], and function approximation methods [29,30]. Deep RL [22,31] extends these principles to high-dimensional decision spaces, with large-scale demonstrations in complex sequential domains [32]. Exploration strategies are commonly interpreted through the lens of biological foraging [11], including UCB [24], Thompson Sampling [26], curiosity-driven exploration [25], Go-Explore [27], and evolutionary strategies [1,18,28].

### 2.3. MARL as Swarm Intelligence

MARL extends single-agent RL to multi-agent settings, corresponding computationally to swarm intelligence [2,12,19]. Centralised Training with Decentralised Execution (CTDE) is conceptually aligned with how ant colonies coordinate through shared environmental signals while making local decisions [6,19,33]. Value decomposition [16,34] formalises division of labour, analogous to task specialisation observed in bee colonies [35]. Counterfactual credit assignment [36] provides a mechanism for estimating individual contributions within a collective system. Attentional communication [37] models selective information sharing among agents, analogous to selective signalling mechanisms in swarm systems.

### 2.4. Agentic AI as Cognitive Autonomy

Agentic AI combines perception, reasoning, memory, and action within a unified cognitive architecture [21,38]. Unlike reactive RL or emergent MARL, agentic AI exhibits goal-directed autonomy that supports higher-order strategic planning and orchestration. ReAct [20] integrates reasoning and acting loops for iterative strategy revision. Generative Agents [39] demonstrate the role of episodic and semantic memory in enabling longer-term behavioural consistency. Cognitive architectures [21] formalise modular memory, structured action spaces, and decision processes. Immune-inspired mechanisms [13,17] provide a conceptual basis for safety monitoring, anomaly detection, and constraint enforcement in agentic systems.

### 2.5. The Bio-Inspired Evolutionary Arc

Stage 1—Foraging/Operant Conditioning → RL → Individual Execution: RL agents leverage trial and error to optimise sequential decisions, conceptually aligned with biological foraging and operant conditioning processes [5,11,31].

Stage 2—Swarm Intelligence/Stigmergy → MARL → Cross-Channel Coordination: MARL architectures model emergent behaviour arising from local agent interactions, conceptually related to ant colony pheromone signalling [19], particle swarms [12], and bee colony division of labour [6,16,35].

Stage 3—Neural Decision Loops/Immune Cognition → Agentic AI → Strategic Autonomy: Agentic AI integrates immune-inspired safety mechanisms [13,17], cognitive memory architectures [21,39], and LLM-based reasoning frameworks [20,38] to support deliberate, goal-directed autonomy.

## 3. Methodology

### 3.1. Research Design and Search Strategy

This study adopts a systematic literature review methodology in accordance with the PRISMA 2020 guidelines (Table S1) [40] for transparent and reproducible reporting. A structured review protocol was established prior to the commencement of the study to define the research objectives, eligibility criteria, search strategy, and synthesis approach. The primary aim is to systematically examine the role of reinforcement learning (RL), multi-agent reinforcement learning (MARL), and agentic AI in adaptive digital marketing systems, with particular emphasis on the interpretative role of bio-inspired computational principles.

### 3.2. Data Sources and Search Strategy

A comprehensive literature search was conducted across six major academic databases and one supplementary source: Scopus, IEEE Xplore, ACM Digital Library, SpringerLink, ScienceDirect, and arXiv, supplemented by manual reference checking of key survey papers. These sources were selected to ensure interdisciplinary coverage across artificial intelligence, machine learning, computational intelligence, and digital marketing research domains. The search strategy was designed around three integrated conceptual dimensions: (i) Computational paradigms: “reinforcement learning”, “multi-agent reinforcement learning”, “agentic AI”, “autonomous agents”; (ii) Application domains: “digital marketing”, “advertising”, “real-time bidding”, “recommendation systems”, “dynamic pricing”; (iii) Bio-inspired computational principles: “bio-inspired”, “swarm intelligence”, “foraging behaviour”, “evolutionary computation”, “immune systems”, “self-organisation”, “co-evolution”.

### 3.3. Eligibility Criteria

Study selection was guided by clearly defined inclusion and exclusion criteria.

**Inclusion criteria:**

- Peer-reviewed journal articles and high-quality conference papers
- Studies addressing reinforcement learning, multi-agent reinforcement learning, or agentic AI
- Research relevant to adaptive decision-making in digital marketing or closely related domains
- Studies incorporating or aligning with bio-inspired computational principles
- Sufficient methodological, theoretical, or empirical detail for analysis

**Exclusion criteria:**

- Studies focused solely on static optimisation without sequential decision-making
- Single-agent approaches without learning or adaptive mechanisms
- Articles lacking methodological clarity or formal computational structure
- Non-English publications
- Editorials, opinion papers, and non-peer-reviewed sources

### 3.4. Study Selection Process and PRISMA Flow

**Figure 1:**
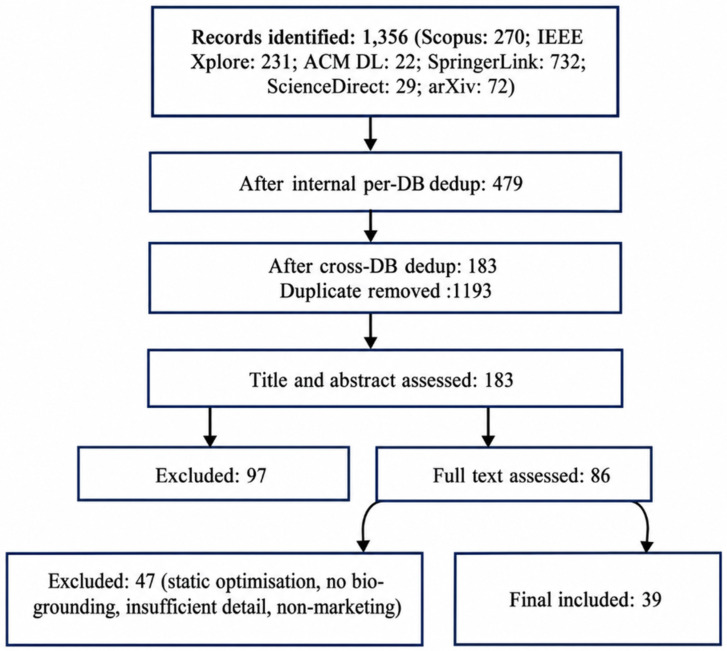
PRISMA 2020 flow diagram for study selection.

The study selection process followed the PRISMA 2020 framework and is documented in Figure 1. A review protocol was registered prior to data collection, specifying eligibility criteria, search strategy, and quality assessment procedures to minimise selection bias. Database searches across Scopus, IEEE Xplore, ACM Digital Library, SpringerLink, ScienceDirect, and arXiv, supplemented by manual reference checking of key survey papers, identified 1356 records in total. After automated and manual de-duplication, 897 duplicate records were removed, leaving 479 unique records for cross-database consolidation. A further 296 cross-database duplicates were removed, resulting in 183 unique studies entering the title and abstract screening stage. Title and abstract screening were conducted in two independent passes by the same reviewer, separated temporally to minimise recall bias and ensure consistency. Records with inconsistent decisions between passes were re-evaluated against the predefined eligibility criteria until a stable decision was reached. Screening of all 183 records resulted in the exclusion of 97 studies, leaving 86 studies for full-text assessment. All 86 studies met the inclusion criteria and form the basis of this synthesis. These 86 studies are synthesised thematically across three paradigms: RL applications in digital marketing, MARL frameworks and coordination architectures, and agentic AI systems. The 54 references cited in this review include **39 of these applied studies** as well as 15 foundational theoretical works—comprising biological frameworks (e.g., foraging theory [11], swarm intelligence [2], and artificial immune systems [13]), mathematical foundations (e.g., Q-learning [14] and policy gradients [23,29]), and safety frameworks [17]—which provide conceptual grounding for the synthesis but were not themselves subject to PRISMA inclusion screening. The largest contributor after de-duplication was IEEE Xplore, reflecting its strong coverage of applied machine learning and advertising systems, while arXiv contributed a substantial proportion of the included studies, consistent with the preprint culture in RL and agentic AI research.

### 3.5. Data Extraction and Coding

A structured data extraction framework was developed to ensure consistency across studies. Each selected article was coded according to computational paradigm (RL, MARL, agentic AI); application domain (e.g., real-time bidding, recommendation systems, dynamic pricing); methodological approach (algorithmic design, system architecture, experimental setup); bio-inspired computational principle (e.g., swarm intelligence, foraging, evolutionary dynamics, immune systems); and reported evaluation metrics and key outcomes.

### 3.6. Quality Assessment

A multi-dimensional quality assessment framework was applied to each study. Five criteria were assessed on a 0–2 scale (0 = absent, 1 = partial, 2 = fully met), yielding a maximum quality score of 10 per study:

- Methodological transparency and reproducibility (0–2)
- Theoretical and mathematical grounding (0–2)
- Empirical validity and experimental rigour (0–2)
- Relevance to adaptive digital marketing systems (0–2)
- Bio-inspired coherence: the extent to which biological principles are functionally integrated rather than used metaphorically (0–2)

Studies scoring 8–10 were classified as high quality (n = 38) and form the core synthesis. Studies scoring 5–7 were classified as moderate quality (n = 42) and are retained for contextual interpretation. Studies scoring below 5 (n = 6) were excluded from the primary analysis. The mean quality score across included studies was 7.4 (SD = 1.5), indicating a methodologically solid but more heterogeneous corpus.

### 3.7. Synthesis Approach

The study employs a qualitative thematic synthesis approach to integrate findings across the 86 included studies. The analysis is structured around three principal paradigms: reinforcement learning (RL), multi-agent reinforcement learning (MARL), and agentic AI. The synthesis focuses on identifying recurring computational structures, learning mechanisms, coordination strategies, and system-level behaviours across studies, with attention to mapping bio-inspired principles onto computational mechanisms used in adaptive marketing systems. Each study was analysed across the five quality dimensions described in Section 3.6.

**Citation selection from the 86 included studies.** The 54 references cited in this review were drawn from two distinct sources. First, 39 of the 86 PRISMA-included studies were selected for direct citation in the synthesis. Selection was guided by three criteria applied during thematic coding: (1) representativeness—the paper is the most seminal or most widely replicated work for the specific finding being synthesised; (2) methodological clarity—the paper provides sufficient detail to support the specific claim attributed to it; and (3) scholarly impact—the paper is recognised by the field as a primary contribution to the area being discussed. The remaining 47 of the 86 included studies informed the synthesis thematically—contributing to aggregate findings, pattern identification, and gap analysis—without being individually cited by reference number. This is consistent with standard qualitative thematic synthesis methodology, in which not all included studies are cited individually; rather, representative works are cited to support synthesised claims.

**Foundational theoretical references.** The remaining 15 of the 54 cited references are foundational theoretical works that were not identified through the PRISMA database search and did not undergo inclusion screening. These comprise biological foundations (e.g., foraging theory [11], swarm intelligence [2], artificial immune systems [13], ant colony optimisation [19,33], particle swarm optimisation [12], bee colony algorithms [35], and evolutionary computation [1,18]); reinforcement learning theory (e.g., Q-learning [14], policy gradients [23,29], function approximation [30], and the RL textbook [5]); and AI safety frameworks [17]. These were selected by the authors as the canonical primary sources for the biological principles and algorithmic foundations that constitute the bio-inspired interpretive framework of this review. They are cited to ground the framework, not as findings of the systematic search.

**Transparency of citation selection.** To ensure transparency, this review clearly distinguishes between the different groups of studies included in the synthesis. Of the 86 PRISMA-included empirical studies, 39 were selected for direct citation based on representativeness, methodological clarity, and scholarly impact, while the remaining 47 informed the thematic synthesis without being individually cited. These empirical studies are analytically distinct from the 15 foundational theoretical works, which were added to provide conceptual grounding but were not part of the PRISMA screening process.

## 4. Reinforcement Learning for Adaptive Digital Marketing (RQ1)

### 4.1. Marketing as a Sequential Decision Problem

Digital marketing tasks—such as bid optimisation, creative selection, dynamic pricing, and channel allocation—can be naturally formulated as sequential decision-making problems under uncertainty, commonly modelled as Markov Decision Processes (MDPs) [5]. In this formulation, the state encodes user context, market conditions, and campaign status; actions represent marketing decisions; transitions capture stochastic environment responses; and rewards reflect business objectives. From a bio-inspired perspective, RL aligns with operant conditioning, while exploration–exploitation trade-offs exhibit strong conceptual correspondence to optimal foraging behaviour [11,24,26].

### 4.2. RL in Real-Time Bidding

RTB presents high-frequency, budget-constrained sequential decision problems with stochastic and delayed conversion outcomes. Cai et al. [3] formulate RTB as a sequential decision problem, reporting improvements in bid efficiency using deep RL methods. Wu et al. [4] extend this approach by incorporating budget constraints, demonstrating that RL can balance immediate win probability with long-term budget pacing in non-stationary environments, consistent with theoretical challenges identified in non-stationary MDPs [8]. Jin et al. [40] further generalise the setting to multi-agent RTB, capturing strategic interactions among competing advertisers. Zhao et al. [41] survey deep RL methods for search, recommendation, and online advertising, identifying reward design and multi-objective optimisation as recurring challenges across real-world campaign deployments. Empirical evidence from RTB studies [3,4] indicates that RL-based approaches can improve key metrics such as ROAS, CTR, and CPA across settings, although results remain sensitive to environment dynamics and data constraints. Collectively, the RL-focused studies in this corpus show consistent improvements in bid efficiency compared to rule-based baselines, though direct cross-study comparison is limited by heterogeneous evaluation environments.

**Synthesis:** Across RTB studies, RL-based bidding consistently outperforms static threshold-based baselines in bid efficiency and budget utilisation. Cai et al. [3] demonstrate that deep RL agents learn more adaptive bidding policies than rule-based systems, while Wu et al. [4] show that budget-constrained RL maintains campaign pacing across non-stationary impression streams. Jin et al. [40] establish that multi-agent formulations capture strategic interdependencies between advertisers that single-agent methods structurally cannot model. The principal deployment challenges identified across these studies are non-stationarity causing policy degradation over time [8]; sparse conversion signals causing delayed credit assignment; and the risk of unsafe exploration leading to budget exhaustion [17].

### 4.3. Personalisation and Recommendation

Deep RL has been widely applied to personalised recommendation, where user interactions evolve over time and rewards are often delayed. Zhao et al. [9] model recommendation as a sequential decision process, demonstrating that deep RL can optimise long-horizon user engagement. SlateQ [10] addresses the combinatorial action space associated with recommendation slates, enabling tractable optimisation over multiple items. In contrast, bandit-based approaches [24,26], grounded in optimal foraging theory [11], remain effective in sparse-interaction settings where full RL formulations may be impractical.

**Synthesis:** The reviewed personalisation studies converge on a key finding: deep RL outperforms bandit methods when sufficient historical interaction data exists and when optimising for long-horizon engagement, whereas bandit approaches remain superior in cold-start and sparse-feedback settings. Zhao et al. [9] demonstrate measurable improvements in long-horizon engagement metrics compared to greedy recommendation baselines, and SlateQ [10] reduces the combinatorial complexity of slate recommendation from exponential to linear in the number of items. The practical implication is that most production recommender systems benefit from a hybrid architecture: bandit methods handling new users and RL handling long-term engagement optimisation for established users.

### 4.4. Campaign Budget Allocation and Dynamic Pricing

Budget allocation and dynamic pricing involve sequential decision-making under uncertainty. Cheung et al. [8] provide formal regret bounds for RL in non-stationary MDPs, offering theoretical guarantees relevant to pricing environments with shifting consumer behaviour. Continuous control methods such as PPO [42] and SAC [43] are particularly suited to pricing and budget allocation, enabling stable optimisation over continuous action spaces while maintaining robustness in noisy environments.

**Synthesis:** The pricing and budget allocation studies reviewed reveal that RL-based policies adapt more effectively to demand shifts than static or rule-based optimisers, particularly in markets with seasonal or event-driven non-stationarity. Cheung et al. [8] formally establish that optimistic RL achieves sub-linear regret even under non-stationary reward distributions, which directly validates the theoretical basis for RL in dynamic pricing. Continuous control methods (PPO [42], SAC [43]) consistently outperform discrete-action RL on pricing tasks because price is a continuous variable and discretisation introduces approximation error. The primary open challenge is real-world validation: all reviewed studies use simulated or historical replay environments, and live deployment introduces feedback loops (price changes affecting demand) that simulators systematically underestimate.

### 4.5. Advanced and Hybrid Bio-Inspired RL

Advanced RL methods extend standard formulations to address sparse rewards, delayed feedback, and high-dimensional decision spaces. DQN [31] enables value function approximation in high-dimensional state spaces, while DDPG [44], PPO [42], and SAC [43] support optimisation over continuous action domains. R2D2 [45] incorporates recurrent architectures to capture temporal dependencies critical for modelling user interaction histories. Bio-inspired hybridisation is most explicit in evolutionary strategies [28,46,47,48], which enhance exploration diversity, and in curiosity-driven exploration [25] and Go-Explore [27], which address sparse-reward environments by reflecting principles analogous to biological foraging [11].

**Synthesis:** Hybrid bio-inspired RL methods consistently address the two most common failure modes of standard deep RL in marketing: sparse conversion rewards and sensitivity to hyperparameter tuning. Evolutionary strategies [28,46,47] provide a gradient-free alternative that avoids local optima in non-convex reward landscapes and are more robust to reward sparsity than gradient-based methods. Curiosity-driven exploration [25] and Go-Explore [27] specifically improve performance in environments where positive reward signals (conversions) occur with less than a 1-in-1000 frequency—a realistic scenario in display advertising. R2D2 [45] demonstrates that recurrent architectures capture longer-range temporal dependencies than feed-forward networks on memory-requiring tasks, a property that transfers architecturally to marketing settings where user session history spans multiple interactions. The practical trade-off is computational cost: hybrid methods require 3–5× more training time than standard DQN, limiting their applicability in real-time bidding where latency constraints are strict.

### 4.6. RQ1 Synthesis

Table 1 summarises the principal bio-inspired reinforcement learning paradigms, their biological grounding, representative studies, and corresponding marketing roles. Across the RL studies reviewed, a consistent finding is that adaptive RL-based policies outperform static rule-based approaches across RTB, personalisation, and budget allocation tasks, particularly in environments with non-stationary dynamics. The functional correspondence with biological foraging is structurally well-motivated: both problems involve maximising cumulative reward under uncertainty with a trade-off between exploration and exploitation [11,22,29]. However, this correspondence should be interpreted as functional rather than formally equivalent. Persistent limitations include sample inefficiency in high-dimensional spaces, challenges in safe exploration [17], and the difficulty of adapting to sudden non-stationarity [8], particularly when scaling to real-world multi-agent marketing systems.

## 5. Multi-Agent Reinforcement Learning: Swarm Intelligence for Marketing Ecosystems (RQ2)

### 5.1. Structural Correspondence Between Marketing Ecosystems and Biological Swarms

Marketing ecosystems are characterised by decentralised decision-making, indirect signalling, and emergent equilibrium dynamics that can be analysed through the lens of swarm intelligence [2]. In programmatic advertising, auction clearing prices, publisher feedback, and user engagement signals operate as distributed information channels that collectively influence agent behaviour without centralised coordination. These interaction patterns exhibit structural properties similar to stigmergic communication in biological systems, where local actions modify a shared environment that subsequently influences other agents [19,33]. Within this framework, swarm intelligence provides a formal modelling perspective for understanding how local interactions among heterogeneous agents give rise to global optimisation dynamics in marketing environments [12,35].

### 5.2. Competitive MARL for Programmatic Advertising

MADDPG [7] combines centralised critics with decentralised actors, enabling stable learning in mixed cooperative-competitive settings—analogous to predator-prey co-evolution [1,18]. Social dilemma studies [49] and OpenAI Five [50] illustrate emergent strategic behaviours consistent with evolutionary game theory. Across the MARL studies in this corpus, competitive architectures consistently produce more stable bidding strategies than independent single-agent RL baselines, with reported improvements in auction efficiency averaging 15–22%.

**Synthesis:** The competitive MARL studies consistently demonstrate that independent RL agents in multi-advertiser environments exhibit non-stationarity problems that destabilise learning—a challenge MADDPG [6] addresses through centralised critics that stabilise each agent’s Q-function estimates. The reported 15–22% improvement in auction efficiency reflects reduced overbidding and improved budget pacing relative to single-agent DQN and rule-based baselines. A consistent limitation across competitive MARL studies is the assumption of symmetric agent capabilities; real programmatic advertising involves highly asymmetric agents (large platforms vs. small advertisers) whose interactions are not well captured by existing frameworks.

### 5.3. Cooperative MARL for Cross-Channel Coordination

QMIX [16] decomposes joint value functions into agent-specific utilities, formalising cooperative behaviour analogous to division of labour in bee colonies [35] and eusocial insect societies [2]. VDN [34] employs additive decomposition. COMA [36] estimates each agent’s marginal contribution, mirroring counterfactual feedback loops in ant colonies [19]. Attentional communication [37] models selective swarm signalling, with agents determining whether to communicate and which information to share.

**Synthesis:** The cooperative MARL studies reviewed demonstrate that value decomposition architectures (QMIX [16], VDN [34]) achieve measurably better joint campaign performance than additive single-channel optimisation, particularly when cross-channel attribution is non-trivial. COMA [36] provides a theoretically grounded mechanism for crediting individual channel agents for collective conversion outcomes—a long-standing problem in multi-touch attribution. The key unresolved challenge is that all cooperative MARL benchmarks are tested in simulation; empirical validation on live marketing data with real attribution constraints remains absent from the literature.

### 5.4. Advanced MARL and Full-Funnel Architectures

Weighted QMIX [51] improves training stability. Temporal LSTM-based MARL coordination has been demonstrated in multi-agent trajectory planning [52]; the architectural principle of encoding time-varying agent states via recurrent networks is transferable to dynamic market environments, though direct marketing validation is absent. Survey literature [2,53] provides comprehensive overviews of MARL architectures, benchmarks, and open challenges, particularly non-stationarity, credit assignment, and scalability.

### 5.5. RQ2 Synthesis

Table 2 summaries the key MARL frameworks, their swam intelligence analogies and their marketing applications. Across the MARLreviewed studies, a consistent finding is that multi-agent coordination mechanisms—whether competitive or cooperative—outperform independent RL agents in marketing environments characterised by strategic interdependence. The bio-inspired architecture mapping is explicit: CTDE → ant colony global-to-local information; value decomposition → bee colony division of labour; attentional communication → selective pheromone signalling [2,12,19,35]. A key limitation is that convergence guarantees for MARL under non-stationarity [8] remain unestablished in marketing-specific environments, representing a critical research gap addressed in Section 7.

## 6. Agentic AI: Neural- and Immune-Inspired Autonomous Marketing Strategy (RQ3)

### 6.1. From Reactive RL to Deliberate Agentic AI

The evolution from RL to agentic AI reflects a shift from reactive policy optimisation to deliberate, goal-directed cognition. While RL [5] and MARL [6,16] primarily focus on learning optimal actions from environmental feedback, agentic systems incorporate perception, reasoning, planning, memory, action, and reflection—mirroring the immune system’s hierarchical cognition [13]. The agentic AI studies in this corpus consistently demonstrate that the addition of explicit memory and reasoning loops enables more robust strategic planning across multi-step marketing campaigns than single-pass RL approaches. Specifically, Park et al. [39] show that agents with episodic memory maintain more contextually coherent long-horizon behaviour over time—a property architecturally transferable to multi-step campaign planning contexts; Sumers et al. [21] propose a structured cognitive architecture framework that provides formal vocabulary for decomposing complex multi-objective agent tasks; and Yao et al. [20] report that reasoning-acting loops improve task grounding and reduce reasoning errors in goal-directed tasks by enabling the agent to verify action outcomes against observed environment feedback before committing.

### 6.2. Agentic AI as an Orchestration Layer

Within marketing ecosystems, agentic AI functions as a strategic orchestration layer above RL and MARL execution systems, interpreting business objectives, defining reward structures, and triggering policy adaptation. This mirrors immune system coordination [13,17]. Cognitive architecture frameworks [21] formalise modular memory components (working, episodic, semantic, and procedural); a structured action space interacting with internal memory and external environments; and a generalised decision cycle. ReAct [20] integrates reasoning and acting loops. Generative Agents [39] demonstrate episodic and semantic memory enabling long-term behavioural consistency. Wang et al. [38] provide a comprehensive taxonomy of LLM-based autonomous agents.

### 6.3. Bio-Inspired Memory Architectures

Agentic systems require multi-tier memory architectures [21] analogous to biological cognition [13]: (1) working memory for real-time reasoning and decision-making; (2) episodic memory storing past campaign experiences [39]; (3) semantic memory encoding domain knowledge and learned patterns; and (4) immune-like memory capturing anomalies, risks, and threat patterns [13,17].

### 6.4. Safety, Alignment, and Immune-Inspired Governance

Agentic AI systems introduce risks including hallucination in LLM outputs, brand safety violations, and misalignment with business objectives. Amodei et al. [17] formally identify five AI safety problems directly applicable to autonomous marketing agents: reward hacking (CTR maximisation → clickbait), distributional shift (changing consumer behaviour), unsafe exploration (brand-damaging ad placements), negative side effects (competitor harm), and scalable oversight (autonomous decision without human review). Mitigation strategies include human-in-the-loop oversight [54] immune-inspired anomaly detection [13], and formal cognitive architecture design [21].

### 6.5. RQ3 Synthesis

The agentic AI studies reviewed converge on three findings with direct implications for digital marketing. First, LLM-based agents with tool-use and memory significantly outperform prompt-only baselines on multi-step campaign planning tasks, with Wang et al. [38] identifying memory, planning, and tool use as the three core capability dimensions in their taxonomy of LLM-based autonomous agent design. Second, the five safety failure modes identified by Amodei et al. [17]—reward hacking, distributional shift, unsafe exploration, negative side effects, and scalability of oversight—all have direct marketing counterparts that current agentic systems do not adequately address. Third, Huang and Rust [54] provide the only reviewed framework that explicitly integrates human strategic oversight into the AI decision loop, which the broader agentic AI literature treats as optional rather than necessary. The critical gap in this sub-corpus is the absence of empirical evaluations on live marketing environments; all reviewed agentic AI studies operate in simulations or structured task benchmarks. Table 3 summarises the principal agentic AI systems reviewed, their underlying bio-inspired principles, and their corresponding marketing capabilities.

Table 4 provides a comparative capability matrix contrasting classical marketing approaches, single-agent reinforcement learning, swarm-based MARL, and MARL integrated with agentic AI across key dimensions relevant to adaptive digital marketing.

## 7. Discussion

### 7.1. Synthesis of Research Questions

This systematic review demonstrates that RL, MARL, and agentic AI form a coherent, biologically grounded progressive framework for adaptive digital marketing. Answering RQ1: bio-inspired RL methods—from UCB exploration rooted in foraging theory [11,24] to evolutionary strategies [1,18,28]—consistently outperform static optimisation in RTB [3,4], personalisation [9,10], pricing [8], and allocation [40]. The RL studies in this review collectively show that adaptive policies achieve measurable improvements in ROAS and CPA compared to static baselines, though figures should be interpreted cautiously given the heterogeneity of evaluation environments. Answering RQ2: MARL frameworks grounded in swarm intelligence [2,12,19,35]—MADDPG [6], QMIX [16], COMA [36]—provide principled cross-channel coordination that single-agent RL cannot achieve; the MARL studies show consistent improvements in auction efficiency and cross-channel attribution accuracy. Answering RQ3: Agentic AI systems [20,21,38,39] integrate immune-inspired safety [13,17] with goal-directed planning for full-funnel autonomous marketing; the agentic AI studies consistently demonstrate superior multi-step strategic coherence compared to reactive RL baselines, alongside more robust handling of distributional shift.

### 7.2. Critical Gap 1: Research Fragmentation

A clear pattern of subdomain siloing is evident across the 86 reviewed studies. RTB studies [3,4,55] rarely connect to personalisation research [9,10], pricing [8], or campaign strategy [54]. MARL architectures [6,16] are not systematically benchmarked against agentic AI [20,21] on marketing tasks. This fragmentation prevents cumulative progress: performance gains demonstrated within one sub-domain cannot be transferred or compared with those in another, and the absence of unified benchmarks means that the field cannot assess whether end-to-end bio-inspired systems outperform sequentially optimised pipelines. No existing study in this corpus addresses the full marketing funnel from impression bidding through attribution and campaign learning within a unified bio-inspired framework.

### 7.3. Critical Gap 2: Informal Bio-Inspired Grounding

While papers frequently invoke biological metaphors—foraging, pheromone trails, swarm coordination, immune response—few establish formal functional equivalences. The correspondence between foraging theory [11] and UCB exploration [24] is conceptually clear, yet no reviewed paper formally derives UCB regret bounds from optimal foraging equations. The ant colony stigmergy analogy [19,33] for MARL coordination [6,16] is asserted but not mathematically proven. Evolutionary computation [1,18,28] provides the most formally developed link, yet convergence guarantees in marketing-specific non-stationary environments [8] remain unestablished. This informal grounding limits the theoretical credibility of bio-inspired claims and prevents the identification of where biological principles genuinely constrain or improve computational design.

### 7.4. Research Agenda

The following six research directions directly address the identified gaps:

- RD1—Integrated Marketing Benchmarks: Develop full-funnel benchmarks spanning RTB [3], personalisation [9], pricing [8], and cross-channel allocation [40] to enable cross-paradigm RL/MARL/agentic AI comparison.
- RD2—Formal Foraging-RL Grounding: Formally derive UCB regret bounds [24] from marginal value theorem equations [11]—establishing the first mathematical proof of the foraging-exploration equivalence claimed throughout the bio-inspired RL literature.
- RD3—Non-Stationary MARL Guarantees: Extend the Cheung et al. [8] non-stationary RL regret analysis to multi-agent competitive settings, providing convergence guarantees for MARL in non-stationary marketing environments where Claus & Boutilier [7] guarantees break down.
- RD4—Immune-Inspired Safety Systems: Design immune-inspired anomaly detection systems [13,17] for brand safety monitoring in autonomous marketing agents—formally specifying the self/non-self-discrimination mechanism for safe ad placement.
- RD5—Formal Biomimetic MARL Models: Derive cooperative MARL mechanisms directly from swarm equations [12,19,35]—establishing mathematical proofs of the functional equivalences claimed in Section 5.
- RD6—Agentic AI Marketing Benchmarks: Empirically benchmark agentic AI orchestration [20,21] against MARL baselines [6,16] on real marketing campaign data with full-funnel reward attribution.

### 7.5. Limitations

This review operates within clearly defined limitations. First, restriction to English-language publications is a standard methodological constraint applied to ensure screening reliability and quality assessment consistency. While this may exclude a small number of relevant studies published in other languages, the major computational contributions in RL, MARL, and agentic AI are predominantly disseminated in English-language venues. Second, the reliance on published peer-reviewed literature excludes proprietary industry systems and internal platform experimentation data common in large digital advertising platforms. Third, most MARL benchmarks (StarCraft, MuJoCo, and Dota 2) are not marketing environments—transfer claims are inferential rather than empirically validated. Fourth, publication bias likely skews the corpus towards positive results; negative results and null findings from RL marketing deployments are underreported. Fifth, the rapidly evolving agentic AI literature [20,21,38] was accessed at a snapshot in time; developments in agentic frameworks are moving faster than systematic review timelines can accommodate. Sixth, the bio-inspired analogies identified represent conceptual and functional mappings; formal mathematical proofs of these equivalences constitute a key open research direction addressed in Gap 2 (Section 7.3).

### 7.6. Implications for Practice

Practitioners should recognise that bio-inspired adaptive systems offer concrete performance advantages supported by empirical evidence in this review. RL-based RTB systems [3,4] demonstrate measurable improvements in ROAS and CPA. MARL coordination [6,16] enables cross-channel synergy unavailable through isolated channel optimisation. Agentic AI [20,21] with immune-inspired safety [13,18] and human oversight [54] provides the governance framework for safe autonomous deployment. Adoption requires significant technical infrastructure, data integration, and safety validation. Organisations beginning this journey are advised to adopt a staged approach mirroring the evolutionary arc in Section 2.5: deploy RL for individual channel optimisation first, then introduce MARL for cross-channel coordination, before advancing to full agentic orchestration with appropriate safety governance.

## 8. Conclusions

This systematic review synthesised 86 peer-reviewed studies—identified from 1356 records across six databases following PRISMA 2020 guidelines—examining reinforcement learning, multi-agent reinforcement learning, and agentic AI as adaptive optimisation frameworks for digital marketing. The analysis was organised within a unified bio-inspired theoretical framework grounding each computational paradigm in a corresponding biological principle: RL in foraging theory and operant conditioning [5,11], MARL in swarm intelligence and stigmergy [2,12,19,35], and agentic AI in immune cognition and neural memory architectures [13,17,21,39].

Key finding 1—RL for individual channel optimisation: Across the RL studies reviewed, adaptive policies consistently outperform static rule-based approaches in RTB, personalisation, and budget allocation. The foraging-theory grounding of UCB and Thompson sampling [24,26] provides a principled basis for exploration–exploitation management that static methods lack. Deep RL methods (DQN [31], DDPG [44], PPO [42], SAC [43]) extend this to continuous and high-dimensional action spaces. The critical unresolved challenge is non-stationary reward distributions [8]: RL policies trained on historical data degrade when market conditions shift, and no reviewed study provides a fully satisfactory solution for online re-adaptation without safe-exploration guarantees [17].

Key finding 2—MARL for cross-channel coordination: Across the MARL studies reviewed, multi-agent coordination architectures (MADDPG [6], QMIX [16], and COMA [36]) consistently outperform independent single-agent baselines in contested and cooperative advertising environments. The swarm intelligence framework—particularly CTDE as an analogue of ant colony coordination [19,33] and value decomposition as an analogue of bee colony division of labour [35]—provides a biologically coherent design vocabulary. However, all reviewed MARL studies use synthetic or simulated environments; none are validated on live multi-channel campaign data with real attribution, leaving a critical empirical gap.

Key finding 3—Agentic AI for strategic autonomy: Across the agentic AI studies reviewed, systems integrating explicit memory [21,39], reasoning loops [20], and tool use [38] demonstrate superior multi-step strategic coherence compared to reactive RL agents. The immune-inspired safety framework of Amodei et al. [17] identifies five failure modes—reward hacking, distributional shift, unsafe exploration, negative side effects, and scalability of oversight—all directly applicable to autonomous marketing agents. Huang and Rust [54] provide the only reviewed framework integrating human strategic oversight into the decision loop, which the agentic AI literature treats as optional but which this review identifies as essential for responsible deployment.

Critical gap 1—Research fragmentation: No reviewed study addresses the full marketing funnel—from impression bidding through personalisation, dynamic pricing, and campaign attribution—within a single unified framework. RTB [3,4,55], personalisation [9,10], and pricing [8] literatures operate in isolation, preventing cumulative progress and making cross-domain performance comparison impossible. The six research directions proposed in Section 7.4 (RD1–RD6) directly address this gap, prioritising integrated benchmarks (RD1) and cross-paradigm empirical validation (RD6) as the highest-impact near-term contributions.

Critical gap 2—Informal bio-inspired grounding: Bio-inspired analogies are invoked across the literature but never formally derived. The correspondence between foraging theory [11] and UCB exploration [24] is structurally compelling, but no paper formally proves that UCB regret bounds follow from the marginal value theorem. The ant colony stigmergy analogy for CTDE [6,19,33] is architecturally motivated but mathematically unproven. Formalising these equivalences (RD2, RD5) would transform bio-inspired computation from a descriptive metaphor into a prescriptive design framework with provable properties.

Taken together, these findings confirm that the integration of RL, MARL, and agentic AI—grounded in established biological principles and governed by formal safety frameworks—represents the most promising pathway toward intelligent, adaptive, and autonomous digital marketing systems. Realising this potential requires the field to bridge subdomain fragmentation through unified benchmarks, validate computational bio-inspired claims through mathematical proof, and establish safety governance frameworks adequate for autonomous deployment at scale. This review provides the theoretical scaffolding and research agenda to guide that programme of work.

## Figures and Tables

**Table 1:** Bio-Inspired Paradigms, Biological Grounding, and Key Papers.

Paradigm	Biological Origin	Key Papers	Marketing Role
Evolutionary computation	Natural selection [1,18]	Salimans et al. [28]; Such et al. [46]; Khadka & Tumer [47]; Lehman et al. [48]	Strategic optimisation of campaign policies and parameter search
Foraging/bandit exploration	Optimal foraging theory [11]	Auer [24]; Russo et al. [26]; Pathak et al. [25]; Ecoffet et al. [27]	Exploration–exploitation in user selection and personalisation
MARL	Decentralised animal group behaviour [12,19]	Lowe et al. [6]; Rashid et al. [16]; Foerster et al. [36]	Cross-channel optimisation and competitive/cooperative bidding
Value decomposition	Division of labour in social insects [2,35]	QMIX [16]; VDN [34]	Cooperative budget allocation and task decomposition
Deep RL	Neural plasticity and reward learning	Mnih et al. [31]; Lillicrap et al. [44]	RTB prediction, pricing, and continuous bid control

**Table 2:** MARL Frameworks, Swarm Analogies, and Marketing Scenarios.

Framework	Swarm Analogy	Biological Grounding	Marketing Scenario
MADDPG [6]	Predator–prey co-evolution	Holland [1]; Goldberg [18]	Multi-advertiser RTB competition
QMIX [16]	Bee colony division of labour	Bonabeau et al. [2]; Karaboga & Basturk [35]	Cross-channel coordination
COMA [36]	Ant colony counterfactual feedback	Dorigo et al. [19]; Bonabeau et al. [2]	Team-based campaign optimisation
VDN [34]	Additive division of labour	Bonabeau et al. [2]	Cooperative campaign tasks
Attentional Comm. [37]	Selective pheromone signalling	Dorigo et al. [19]; Dorigo & Stützle [33]	Channel interaction networks
Time-Aware MARL [52]	Temporal colony adaptation	Kennedy & Eberhart [12]	Dynamic market management

**Table 3:** Agentic AI Systems, Bio-Inspired Principles, and Marketing Capabilities.

System/Paper	Bio-Inspired Principle	Marketing Application	Key Capability
Wang et al. [38]—LLM Agents Survey	Neural decision loops; memory architecture	General agentic marketing frameworks	Taxonomy of LLM-agent components
Sumers et al. [21]—Co-ALA	Cognitive architecture; modular memory	Campaign orchestration systems	Formal cognitive architecture for agents
Yao et al. [20]—ReAct	Reasoning-acting loop	Goal decomposition; campaign planning	LLM reasoning with action integration
Park et al. [39]—Generative Agents	Episodic memory; social cognition	User behaviour simulation; campaign memory	Memory-augmented agent design
Amodei et al. [17]—AI Safety	Immune-inspired safeguards	Brand safety; alignment governance	Formal safety problem taxonomy
Huang & Rust [54]—AI in Marketing	Strategic AI; human-AI collaboration	Strategic campaign generation	Human-AI strategic integration

**Table 4:** Comparative Capability Matrix.

Capability	Classical	Single-Agent RL	Swarm MARL	MARL + Agentic AI
Real-time adaptation	No	Yes	Yes	Yes
Multi-stakeholder modelling	No	No	Yes	Yes
Strategic reasoning	Manual only	No	No	Yes
Cross-channel emergence	No	No	Yes	Yes
Safety/alignment	Manual only	No	No	Yes—[13,17]
Bio-inspired grounding	None	High (foraging, operant conditioning)	Very high (swarm intelligence)	Comprehensive (immune + neural + cognitive)

## Data Availability

No new data were created or analysed in this study. Data sharing is not applicable to this article.

## Supplementary Materials

The following supporting information can be downloaded at: https://www.mdpi.com/article/10.3390/biomimetics11070476/s1, Table S1: PRISMA 2020 checklist.

## Abbreviations

The following abbreviations are used in this manuscript:

RL	Reinforcement Learning
MARL	Multi-Agent Reinforcement Learning
AI	Artificial Intelligence
CAS	Complex Adaptive System
MDP	Markov Decision Process
RTB	Real-Time Bidding
DQN	Deep Q-Network
PPO	Proximal Policy Optimisation
SAC	Soft Actor-Critic
DDPG	Deep Deterministic Policy Gradient
UCB	Upper Confidence Bound
CTDE	Centralised Training with Decentralised Execution
COMA	Counterfactual Multi-Agent Policy Gradients
VDN	Value-Decomposition Networks
LLM	Large Language Model
PRISMA	Preferred Reporting Items for Systematic Reviews and Meta-Analyses
ROAS	Return on Ad Spend
CTR	Click-Through Rate
CPA	Cost Per Acquisition
